# 

**Published:** 1881-03

**Authors:** 


### CONTENTS:

ART. I.—Proceedings of Maryland and District of Columbia Dental Association. Annual Meeting.—(Continued.)......481
ART. II.—Pivot Teeth. By Charles L. Steel, D. D. S.......490
ART. III.—Address to the Graduating Class of the Balto. College Dental Surgery. By Rev. Wm. Kirkus, M. A., LL. B.......497
ART. IV.—Feeding of Nursing Children. By Ephraim Cutter, M. D.......504
ART. V—Our Dental Literature. By Prof. L. Ashley Faught, D. D. S.......510
ART. VI.—Heveenoid.—The Rubber of the Future. By Prof. Hy. A. Mott, Jr......513
ART. VII.—Cohesive and Non Cohesive Golds—Correspondence.......518
The Texas State Dental Association.......520

### EDITORIAL, ETC.

The Forty-first Annual Commencement of the Balto. College of Dental Surgery.....520
The late Session of the Baltimore College of Dental Surgery.......523
The Publishers of the American Journal of Dental Science.......524

### BIBLIOGRAPHICAL.

Journals.......525

### MONTHLY SUMMARY.

A Curious Fracture of Skull and Face.......526
Therapeutical Effects of Chlorate of Potash.......527
Adulterations in a New Sugar.......528

